# Motivational Factors for Breast Cancer Screening Behaviors in Iranian Women: A Qualitative Study

**DOI:** 10.31557/APJCP.2020.21.10.3109

**Published:** 2020-10

**Authors:** Hossein Safizadeh, Narjes Amirzadeh, Parvin Mangolian Shahrbabaki

**Affiliations:** 1 *Department of Community Medicine, School of Medicine, Research Center for Social Determinants of Health, Institute for Futures Studies in Health, Kerman University of Medical Sciences, Kerman, Iran. *; 2 *Medical Doctor, Kerman, Iran.*; 3 *Nursing Research Center, Kerman University of Medical Sciences, Kerman, Iran.*

**Keywords:** Motivation, breast cancer, screening, women, qualitative study

## Abstract

**Background:**

Early diagnosis of breast cancer increases the chance of recovery and life expectancy. Screening is the primary tool for early diagnosis and timely treatment of breast cancer in early stages. This qualitative study aimed to explain the motivational factors for breast cancer screening in Iranian women.

**Methods:**

This qualitative study was conducted using content analysis. The 45 women were selected through purposive sampling. Focus group were used for data collection and the data were analyzed using the Lundman and Graneheim thematic content analysis approach.

**Results:**

Data analysis identified 9 themes: knowledge acquisition, presence of happy-hopeful spirit, positive attitude and self-worth, maternal role, intellectual and financial independence, religious beliefs, motivational fears, and supportive family.

**Conclusion:**

According to the results of this study, it seems that health systems need to change the attitudes and beliefs of people to enhance health culture by identifying women’s motivations for breast cancer screening in different groups and increase social knowledge about screening methods by supporting and training.

## Introduction

Breast is considered as a common site for potentially fatal malignancies in women (Malani, 2012). Millions new cases and more than 450,000 deaths occur from breast cancer worldwide per year (Warner, 2011). Early diagnosis of breast cancer is very important timely diagnosis and appropriate treatment increase the chance of recovery and life expectancy (Khanjani et al., 2012). In current medical science, screening is the primary tool for early diagnosis and timely treatment of breast cancer in early stages (Enjezab et al., 2004).

Breast self-examination and clinical examination are accessible, inexpensive, non-invasive method learned and practiced by the individual (Safizadeh et al., 2018). A study showed that cancer-related mortality rates were about 20.5 percent lower in women who were regularly examined by a physician than those who were not examined (Abedian et al., 2006).Mammography is one of the effective methods for detecting breast cancer in asymptomatic women and is complementary to the clinical examination of the patient (Burkman, 2012). Prospective studies have shown a 40% reduction for the second, third and fourth cancer stages with a 30% survival increase in the population screened with mammography (Mohammadifard et al., 2019). Mammography is more accurate than clinical examination and provides a true positive rate of 90% (Alirmayi et al., 2009). It should be noted that the most effective method is a combination of different methods (Safizadeh et al., 2018).

Motivation plays an important role among many different personal and environmental factors affecting health behaviors (Hakim et al., 2015). Motivation plays a very important role in explaining the cause of behavior, predicting the effects of tasks, and guiding the behavior to achieve the goal (Ålgars et al., 2015). Individuals with intrinsic motivation will organize from within and do not allow others and external factors to influence their performance (Lee et al., 2019). Appropriate selection of breast cancer screening-related measures and activities is possible by identifying women’s motivations and removing barriers.

The prevalence of breast cancer is increasing in Iran. The age of breast cancer among Iranian women is almost 10 years compared to developed countries (Monfared et al., 2017). In Iran, concerning the cultural conditions and sensitivity of some organs such as the breast, most women and even young girls ignore breast cancer screening and sometimes do not screen at all and thus breast cancers are diagnosed at an advanced stage and cause a great deal of suffering for women and their families (Safizadeh et al., 2018).

The breast cancer screening is very important and preventive and health beliefs and behaviors in any society are shaped by the social and cultural context of the individuals. This qualitative study aimed to explain the motivational factors for breast cancer screening in Iranian women.

## Materials and Methods

*Study design*

This qualitative research is a content analysis with a conventional approach. Content analysis is the best way to examine participants’ perception and experiences. One of the benefits of this approach is to obtain direct information without imposing predetermined classes, and the researchers have immersed themselves in the data to gain a deeper insight into a phenomenon (Graneheim and Lundman, 2004).

*Sample and setting*

In this study, a total of 45 participants were selected using purposive sampling strategy from four health centers in Kerman located, which are the largest referral centers of health care in the southeast of Iran. These centers were located in different areas and covered people with different economic and social conditions. They were divided into 9 groups of 5 individuals. They only had the minimum of bachelor’s degree and teaching experience in educational centers and had good mental health and were fluent in Farsi. The participants were chosen with a diversity of age, work experiences and education level. The place of the interview was in the health centers of Kerman. Sampling continued until saturation. Saturation means that no new data must not be added to the data.

*Data collection*

Focus group was used for data collection. Focus groups can provide rich information on a specific topic. In each focus group, one of the researchers was responsible for conducting the interviews, and the other was responsible for taking notes. Interviewer encouraged all participants to engage in discussion, share experiences in each 60- to 150-minute semi-structured interview session. Examples of questions were: “Based on your experience, what motivates women to screen for breast cancer? How can such motivations be enhanced? The second researcher took notes about answers based on the number of the participant regarding nonverbal communication such as facial expressions and hand movements.

This study was approved by the Ethics Committee of Kerman University of Medical Sciences (No. 97000297). The researcher informed the participants about the purpose of the study, the confidentiality of the data, and the right to enter and withdraw from the study. The researcher obtained written informed consent from all participants before the interviews. It was emphasized that each participant was responsible for keeping information confidential. Each interview was recorded by a digital recorder with their consent.

*Data analysis*

The process of data analysis was carried out according to the steps proposed by Lundman and Graneheim. For this purpose, each interview was typed verbatim, was read several times to understand the general content, and semantic units and initial codes were determined, then codes were merged and categorized into broader categories according to similarity, and ultimately the hidden concept and content of the data were extracted (Graneheim and Lundman, 2004).

*Accuracy and stability of data*

Guba and Lincoln criteria were used to ensure the accuracy and stability of the data. To ensure the scientific accuracy and reliability of the data, the researcher engaged in ongoing research, data, and participants during one year and provided an in-depth interview with trust-building. A portion of the text along with the original codes was checked by the participants to compare extracted ideas and the concepts and classes developed by skilled observers and experts were reviewed and approved.

## Results

The mean age of participants was 47 years, majority of them were married (92.8%) and were teaching in girls’ schools with a bachelor’s and master’s degree as teachers. 17.8% experienced breast self-examination, 46.4% experienced clinical examination and 42.8% experienced mammography. Data analysis identified 9 themes: knowledge acquisition, presence of happy-hopeful spirit, positive attitude and self-worth, maternal role, intellectual and financial independence, religious beliefs, motivational fears, and supportive family.

*Knowledge acquisition*

One of the most important issues that most participants emphasized as a strong motivation was the acquisition of knowledge about breast cancer and various screening methods that made women aware of breast cancer and the importance of timely diagnosis. Knowledge acquisition made women better understand the importance of the subject and perform it more effectively. Participants emphasized that the more information available on screening methods, the greater the trust and confidence of the individual in screening. With the development of public education through the media and newspapers and the increased level of health knowledge, more women are being encouraged to screen for breast cancer. Most of them emphasized that young girls ‘education and improvement of knowledge level must have been started from high school. A forty-two-year-old woman with said: *“I do breast self-examination, because I accidentally read a brochure about breast cancer and how to do breast cancer self-examination*.”

*The happy- hopeful spirit*

Participants’ experiences indicated that improving self-care behaviors and organizing many health-related behaviors and habits, including breast cancer screening were associated with vitality, happiness and positive emotions. Most participants considered promotion of correct happiness, awareness of people for perception of the real happiness, implementation of appropriate cultural and artistic programs, implementation of public sports programs, creation or improvement of the infrastructure needed to enhance the public happiness, as well as enhancement of indigenous culture as appropriate ways to promote happiness and hope. A thirty eight-year-old woman said: “*One of my friends does care behaviors very carefully, she is regularly examined and does breast self-examination monthly. Her main reason is that she is very happy, does exercise and enjoy leisure time*”.

*Positive attitude and self-worth*

According to the participants’ experiences, positive though and attitude are a very important motivation for promoting health-related behaviors and they emphasize that positive thought had a tremendous impact on one’s personality, health, energy and creativity. A positive attitude can make people value their self and perform health behaviors well. Most participants acknowledged that if people had chosen a positive attitude as an effective way in their life, it would have brought about constructive change in their lives. They believed that, sense of self-worth helps that they trust their own judgment and make better decisions. When their self-worth is higher, they not only feel better about themselves, but they are also sensitive to their health. A forty eight-year-old woman said: “*positive attitude has helped me not to be indifferent to my body, I do screening behaviors as recommended by my doctor*.”

*Maternal role*

Most participants acknowledged that due to the role of motherhood, affection, and attachment to their children, and the great responsibility they have for caring for their children. They preferred to take care of their own health so that they could meet their children’s needs, because, within many families much of the daily mother’s responsibility was keeping the family healthy. They believed that, a healthy and happy mother is a better mother. A fifty three -year-old woman said: “*My only motivation to keep myself in good health is my children, I’m a mother, and if I become sick, my whole family will be hurt*.”

*Intellectual and financial independence*

Many participants considered intellectual independence as an important factor in promoting health behaviors, including breast cancer screening. They believed that an intellectually independent person had the ability to problem-solve and make the right decisions about her life and health. Many participants also considered financial independence to promote women’s health behaviors, in addition to intellectual independence. They emphasized that women’s economic independence made them more prosperous in life and they did not require others in terms of many personal and medical costs. They could use their property for their own health without their spouse’s permission. A forty one-year-old woman said: “*One of the reasons I get examined regularly is that I am a teacher, I have monthly salary, I have golden insurance, my monthly salary makes me confident and I’m not worried about the cost.*”

*Positive religious beliefs*

Many participants believed that religion was a powerful force and that religious behaviors and beliefs had a positive effect on making life meaningful. Person’s positive beliefs are a strong influence for good on their health. The effect of attitudes beliefs on health culture is vast. It affects perceptions of health, illness, beliefs about causes of disease, approaches to health promotion, how illness and pain are experienced and expressed. Having meaning and purpose in life encourages people to engage in health-related behaviors. Religious beliefs increase hope and positive attitudes in life. A thirty one-year-old woman said: “*I love that I am a human with a divine spirit, I have to be physically healthy, thank God that I’m healthy and I can help others, so I have to take care of myself.*”

*Motivational fears*

Participants’ experiences showed that in many cases the fear of cancer and the painful treatment encouraged them for breast cancer screening. Dealing with breast cancer patients or listening to their experiences of chemotherapy and surgery simulated them for screening. A fifty one -year-old woman said: “*A friend of mine was diagnosed with advanced stages of cancer, got chemotherapy, lost all her hair, suffered a lot and died very soon. I’ve done check-up since then, I don’t want to die like her.*”

*Supportive family*

Most participants considered the family as an important and influential factor in their caring behaviors. They believed that if the relationship between mother and children, and especially the couple had been intimate and full of love and interest, their health behaviors would have improved. Emotional relationship between husband and wife and other family members creates happiness, life expectancy and self-efficacy in the family members, they are sensitive to each other’s health and encourage and support one another in health behaviors. A thirty nine -year-old woman said: “*If my husband did not support me, I might not have done check-up. I am satisfied with my family and there is a good emotional relationship between us.*”

**Figure 1:**
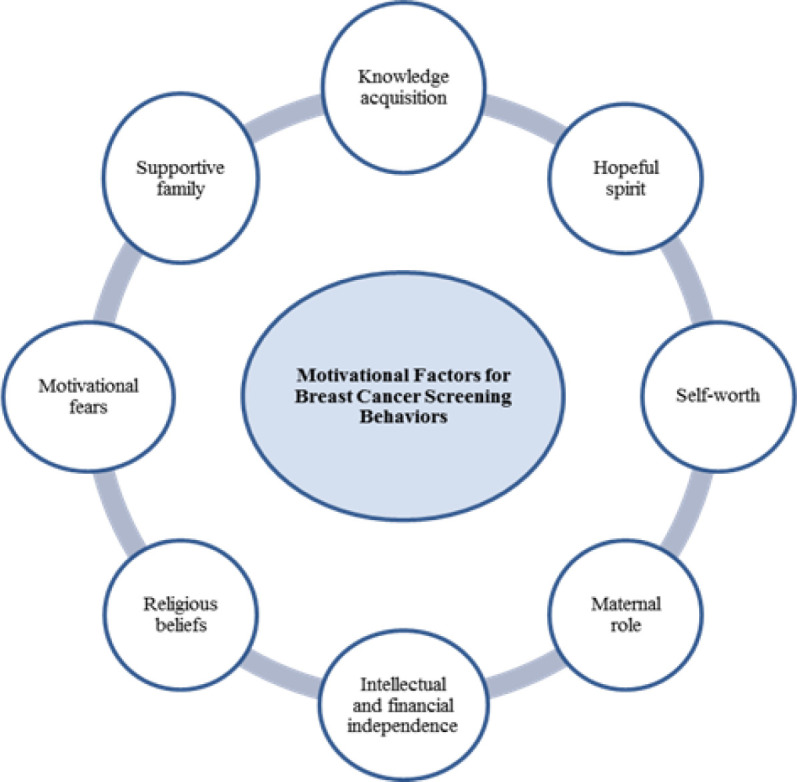
Model of Motivational Factors for Breast Cancer Screening Behaviors

## Discussion

The findings of this study showed that women in south-east of Iran have many motivations for breast cancer screening behaviors. These motivations include knowledge acquisition, presence of happy and hopeful spirit, positive attitude and self-worth, maternal role, intellectual and financial independence, religious beliefs, motivational fears, and supportive families.

Acquiring knowledge through various means such as media and educational classes can lead women to timely breast cancer screening, as knowledge opens new horizons for women and informs them of the benefits of early diagnosis and risks of late diagnosis. In support of these results, A study showed that educated women or those whose husbands were educated might do breast self-examination compared to other women (Rezabeigi-davarani et al., 2016). The most important reason for women’s involvement in breast cancer screening program is to be aware of its importance (Soltanahmadi et al., 2010). 

The spirit of happiness and hope for the future is a very strong motivation for screening among Iranian participants. Because the creativity and flourishing of the talents depend on happiness and vitality, and the human mind is dynamic and its behaviors are logical and effective in a happy environment. Structural changes such as economic, social and political issues, as well as the transition from traditional society to modern society and the world of technology, have led to the emergence of socially various phenomena that have made the community happier than ever before. In line this study, there was a significant positive relationship between Turkish women’s life expectancy and health, and the higher the women’s hope, the higher their health (Nasiri et al., 2012). It seems that hope increased women’s satisfaction and quality of life and made them sensitive to their health. Another study showed that there was a significant positive relationship between hope and self-efficacy and the more hopeful the individuals, the higher their self-efficacy, as well as the better their life styles and health (Anderson and Feldman, 2019).

Positive thought and self-worth are important motivations for breast cancer screening because a positive attitude is a powerful mental state that promotes good and excellent values. Positive thinking and self-worth make people more sensitive to their health and they choose the right lifestyle and plans for health, including breast cancer screening. Based on a study, self-acceptance was highly correlate with self-confidence, and the higher the self-acceptance in individuals, the higher their self-efficacy, the better their self-care planning and the less physical and psychological problems they had (MacInnes, 2006). Based on another study, there was a close relationship amongst self-compassion, self-esteem, and well-being, and the more the people loved themselves, the more confident they were and their behaviors made them feel great (Neff, 2011). It seems that, self-worth can help them the confidence to self-care. Self-care is imperative in maintaining a healthy relationship with yourself and others. It produces positive feelings, which builds self-love and self-confidence.

Maternal role is one of the important motivations that led women to perform breast cancer screening behaviors. Because most women are interested in being healthy and having more opportunity to take care of their children. The mother is the most important part of the family. Her health affects the health of her family and all members of her family would be affected if she became ill. A number of studies have suggested that role playing can be an important motivational factor for promoting correct behaviors and that cultural and educational measures need to be taken to enhance the women’s sense of competence, self-esteem and satisfaction with the maternal role (Mercer, 2004, Ngai et al., 2010). It seems that, Mothering can lead to a sense of fulfilment and meaning so mothers try to take good care of themselves so that they can fulfill their motherly role.

Intellectual and financial independences are other categories that have a major impact on the acceptance of screening behaviors, including breast cancer. Because she freely decides about her own health and freely spends money on her health-related behaviors. Financial support can play a role in building confidence in women and alleviating the fear of no financial support for caring and diagnostic services. Independence is a powerful ideal that allows her to argue that women must be able to act on their own terms as social and political equals (Halldenius, 2015).

Religious beliefs are one of the motivational factors in persuading Iranian women to pay attention to their physical health. Religious beliefs ensure people that a strong force will always support them. These people take events easier by relying on their faith and belief, and they are more hopeful and optimistic about the future. It can also be said that when one is supported by sources of support, they will have a more optimistic view on their life and the future. Some of findings suggest a number of promising research directions on the religion–mental health connection among older Americans (McFarland, 2009). It seems that religious belief lessens depression and promotes self-esteem. Religious belief also increases self-care and lessens the incidence of many diseases.

Motivational fears are among factors that have led Iranian women to do breast cancer screening. A study showed that dealing with breast cancer patients’ experiences could encourage women to do appropriate screening behaviors for early diagnosis of breast cancer (Safizadeh et al., 2018). If women with cancer had talked about their painful experiences of late diagnosis to breast cancer, it would have been effective in encouraging other women to screen for breast cancer. Some of findings confirmed that the most important factors affecting knowledge and practice in breast cancer screening behaviors were family history of cancer, and fear of cancer (Norizadeh et al., 2010).

Supportive families played an important role in Iranian participants’ tendency toward self-care behaviors, including breast cancer screening. In support of these results, a number of authors have shown that emotional relationship amongst family members created happiness and life expectancy and encouraged and supported them in promoting health behaviors (Sebern and Riegel, 2009; Mayberry and Osborn, 2012). It seems that, a familial support system can be between immediate family members, or a mix between extended and immediate family members. Healthy, supportive families typically take care of each other.

The study has limitations common to many qualitative studies. The volume of data makes analysis time-consuming and the presence of researchers during data collection can influence the response of participants, both of which are often unavoidable in qualitative research. Although the limited number of research participants may restrict its generalization. However, it has been attempted to avoid this bias, by deeply describing the results and reviewing all participants’ experiences. 

In conclusion, according to the results of this study, it seems that there are many motivations for regular screening of breast cancer due to the increasing prevalence of breast cancer in Iranian women, but the rate of tendency toward screening is not satisfactory. Therefore, not only physicians but also other health care providers such as nurses should inform women of the role of different screening methods in the timely diagnosis and early treatment of breast cancer. Health systems need to change the attitudes and beliefs of people to enhance health culture by identifying women’s motivations for breast cancer screening in different groups and increase social knowledge about screening methods by supporting and training. Given the cultural differences, it is suggested to conduct further studies in other communities to better understand the barriers and facilitators of breast cancer screening.
